# αvβ3-targeted sEVs for efficient intracellular delivery of proteins using MFG-E8

**DOI:** 10.1186/s12896-022-00745-7

**Published:** 2022-05-21

**Authors:** Junxin Mai, Kai Wang, Chenxuexuan Liu, Sheng Xiong, Qiuling Xie

**Affiliations:** 1 College of Life Science and Technology, Jinan University, Guangzhou, 510632 China; 2 National Engineering Research Center of Genetic Medicine, Guangzhou, 510632 China

**Keywords:** Small extracellular vesicles (sEVs); milk fat globule–epidermal growth factor 8 protein (MFG-E8), αvβ3 integrin target, Drug delivery

## Abstract

**Background:**

Small extracellular vesicles (sEVs) are nanometer-sized membranous particles shed by many types of cells and can transfer a multitude of cargos between cells. Recent studies of sEVs have been focusing on their potential to be novel drug carriers due to natural composition and other promising characteristics. However, there are challenges in sEVs-based drug delivery, one of which is the inefficient loading of drugs into sEVs, especially for large biomolecules.

**Results:**

In this study, we proposed a membrane-associated protein, milk fat globule–epidermal growth factor 8 protein (MFG-E8), to produce αvβ3-targeted sEVs with high delivery efficiency of interested protein. MFG-E8 is a secreted protein with NH2-terminal epidermal growth factor (EGF)–like domains, containing an Arg-Gly-Asp(RGD) sequence that binds αvβ3 and αvβ5 integrins, and COOH terminal domains C1 and C2, which can bind to lipid membrane with strong affinity. Firstly, we transiently expressed MFG-E8 in HEK293F cells and found that this protein could be secreted and adhere to the cell membrane. The recombinant MFG-E8 is also found to locate at the outer membrane of sEVs. Then we generated engineered sEVs by expressing high levels of the EGFP fused to MFG-E8 in HEK293F cells and showed that MFG-E8 could increase the delivery efficiency of EGFP into sEVs. Further delivery of Gaussia luciferase (GL) by fusion expression with MFG-E8 in donor cells demonstrated that target proteins fused with MFG-E8 still kept their activity. Finally, we identified the sEVs’ target to integrin αvβ3 by comparing the transfection efficiency with MFG-E8 loaded sEVs (MFG-E8-sEVs) in αvβ3 positive cells and αvβ3 negative cells. Analysis showed higher target protein could transfect into αvβ3 positive cells with MFG-E8-sEVs than with EGFP loaded sEVs (EGFP-sEVs), meaning the engineered sEVs with MFG-E8 not only could increase the delivery of target protein into sEVs, but also could target the αvβ3 positive cells.

**Conclusion:**

This study suggests that recombinant MFG-E8 is an ideal protein to increasingly deliver the drug into sEVs and give sEVs the ability to target the αvβ3 positive cells.

**Supplementary Information:**

The online version contains supplementary material available at 10.1186/s12896-022-00745-7.

## Background

Small extracellular vesicles (sEVs) are membranous vesicles with 40–120 nm in diameter released by a variety of cells. They are thought to play a key role in cell-to-cell communication by transporting a multitude of cargos between cells, including mRNAs, proteins, microRNA (miRNA), non-coding RNAs, and DNA, impacting many physiological and pathological cellular processes, such as immune response, inflammation, cancer progression, and et al. [1–8]. sEVs have also been detected as diagnostic, prognostic, and treatment monitoring biomarkers [9, 10]. In recent years, sEVs have been studied as potential therapeutic agents and viable vaccines in clinical immunotherapy [6, 10–13].

sEVs also have the potential to be drug delivery vehicles because of their natural composition. Compared with other nanoparticles such as liposomes or polymeric nanoparticles, sEVs are superior in that: 1. They have low immunogenicity due to their small size and the same bilayer cellular membrane as human cells. 2. They have high permeability to migrate through various biological barriers, such as mucosal and blood–brain barrier. 3. They are more stable than artificial nanoparticles in the circulation system because they can bypass complement activation to avoid phagocytosis and degradation. 4. Furthermore, the loading of hydrophobic compounds into sEVs was found to be higher than in liposomes [14–17].

sEVs have been exploited for therapeutic drug delivery as seen in tumor chemotherapeutic agents including curcumin [18–20], doxorubicin (Dox) or paclitaxel (PTX) [21, 22]. The capability of delivering exogenous RNAs, especially siRNA, has also been under several investigations [23–25]. Currently, there are two different approaches for loading drugs into sEVs: exogenous (i.e. after sEVs isolation) and endogenous loading (i.e. during sEVs biogenesis) [26]. For exogenous loading of sEVs, different techniques have been employed, including incubation at room temperature, permeabilization with saponin, freeze–thaw cycles, sonication, or extrusion [27–29]. However, these techniques could result in the aggregation of sEVs or their cargo and even alteration of their physicochemical or morphological characteristics [30]. Moreover, these aforementioned technics are less promising for functional proteins because of their larger molecular weight [31]. On the other hand, the endogenous approach is more suitable for protein loading, where sEVs can be loaded during biogenesis via direct transfection of a recombinant vector with genes of interested protein. After synthesized, the recombinant protein is sorted into sEVs with other cytosolic constituents.

Because the sorting mechanism of cytosolic protein into sEVs is poorly understood, a strategy of efficient loading is to fuse the therapeutic protein with proteins enriched in sEVs, such as CD63, CD9, et al.[31–34]. In this report, we proposed a membrane-associated protein milk fat globule–epidermal growth factor 8 protein(MFG-E8) to deliver proteins into sEVs. MFG-E8 is a secreted protein with three functional domains: NH2-terminal epidermal growth factor (EGF)–like domains, which contain an Arg-Gly-Asp sequence that binds αvβ3 and αvβ5 integrins, and COOH terminal domains C1 and C2, which can bind to lipid membrane with strong affinity [35, 36]. MFG-E8 was also found to be abundant in sEVs secreted by many kinds of cells [37, 38]. We sought to transiently express the exogenous proteins by fusion them with MFG-E8 in HEK293 cells and to dress exogenous proteins onto sEVs with the C1C2 domain of MFG-E8. Meanwhile, we verified the (EGF)–like domains could target the sEVs to cells with overexpression of αvβ3 integrins.

## Results

### Recombinant MFG-E8 secreted from host cells but retained outside of cells

**Fig. 1:**
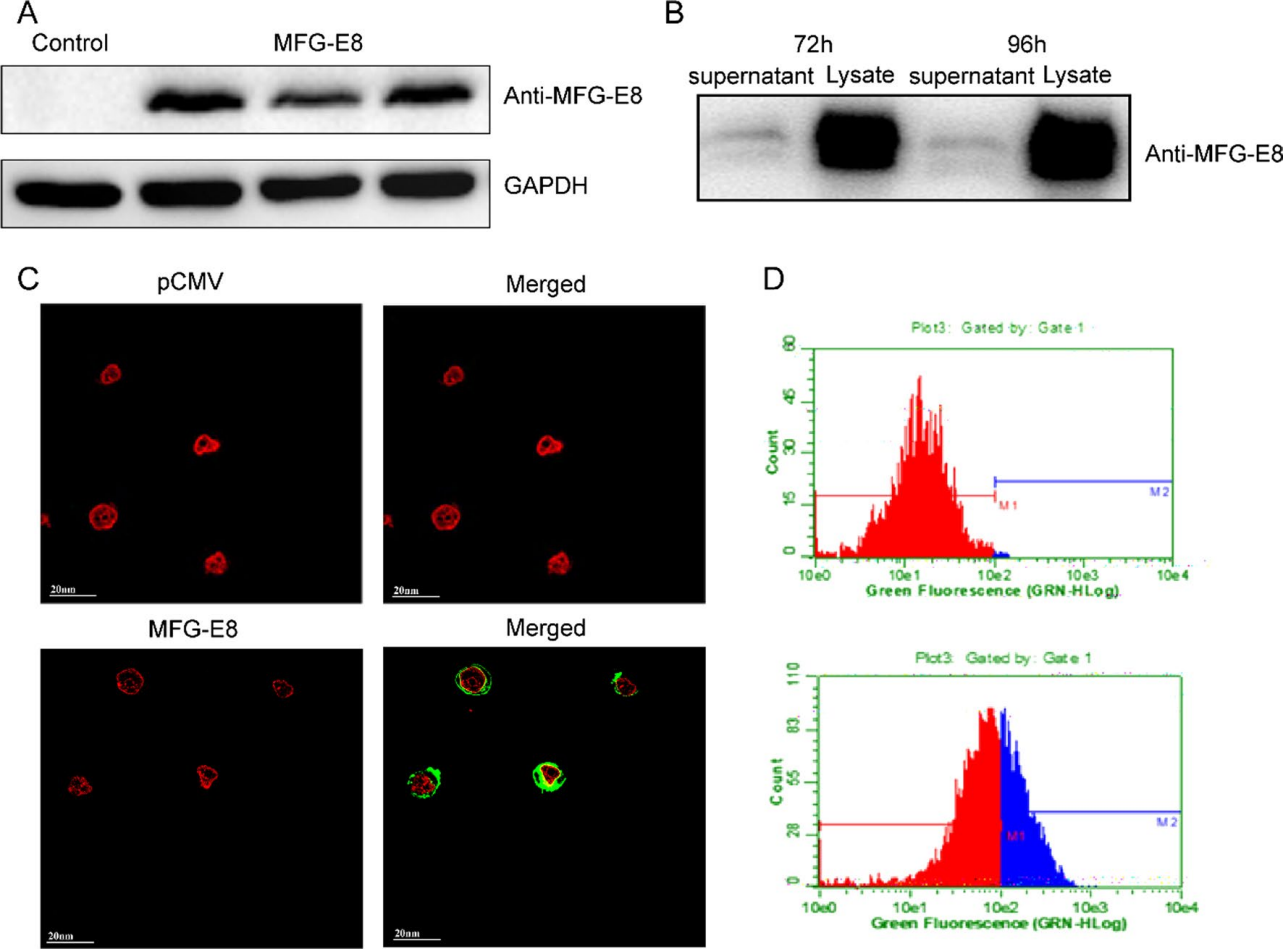
Transient expression of MFG-E8 in HEK293F cells. HEK 293F cells were transfected with pCDNA 3.4/MFG-E8, and the expression of recombinant MFG-E8 was confirmed by western blotting (**a** and Additional file 1: Figures S1, S2). After centrifugation of cell culture, the recombinant protein was found in precipitate but not supernatant (**b** and Additional file 1: Figure S3). The recombinant MFG-E8 was further confirmed to link with outside of cell membrane with anti-MFG-E8 antibody by laser confocal microscope (**c**) and flow cytometry (**d**). In the laser confocal microscope, the red stain refers to the cell membrane, and the green stain refers to the MFG-E8

The recombinant plasmid with an MFG-E8 protein-coding gene and a signal peptide sequence was constructed and transfected into HEK293F cells to transiently express the recombinant MFG-E8. On day 4 of post-transfection, cell culture was harvested and analyzed by western blotting. MFG-E8 could be expressed in 293F cells successfully (Fig. 1a). However, most of the proteins existed in the cell debris with few found in the supernatant of cell culture, meaning few proteins secreted outside the host cells despite MFG-E8 having a signal peptide in the N-terminal (Fig. 1b).

Because MFG-E8 contains C1 and C2 domains which can bind to the lipid of the cell membrane [35, 36], we sought to confirm if recombinant MFG-E8 adhered to the cell membrane. We incubated the cells with anti-MFG-E8 antibody and anti-Mouse IgG H&L (FITC) (green) as a second antibody, while using DIL for the cell membrane (red). We analyzed the mixture by flow cytometry and laser confocal microscope. It was found that MFG-E8 protein (green) was located outside the cell membrane under a confocal microscope (Fig. 1c) and about 40% of the cells were FITC-positive indicated by flow cytometry analysis (Fig. 1d). The results above showed that the recombinant MFG-E8 was secreted from the cell but  attached to the outside of the cell membrane.

### MFG-E8 could enter sEVs and linked to outside of sEVs membrane

The sEVs were isolated from the supernatant of cell culture by successive ultracentrifugation at increasing speeds, followed by multiple times of washing to further eliminate the contaminating proteins.

**Fig. 2:**
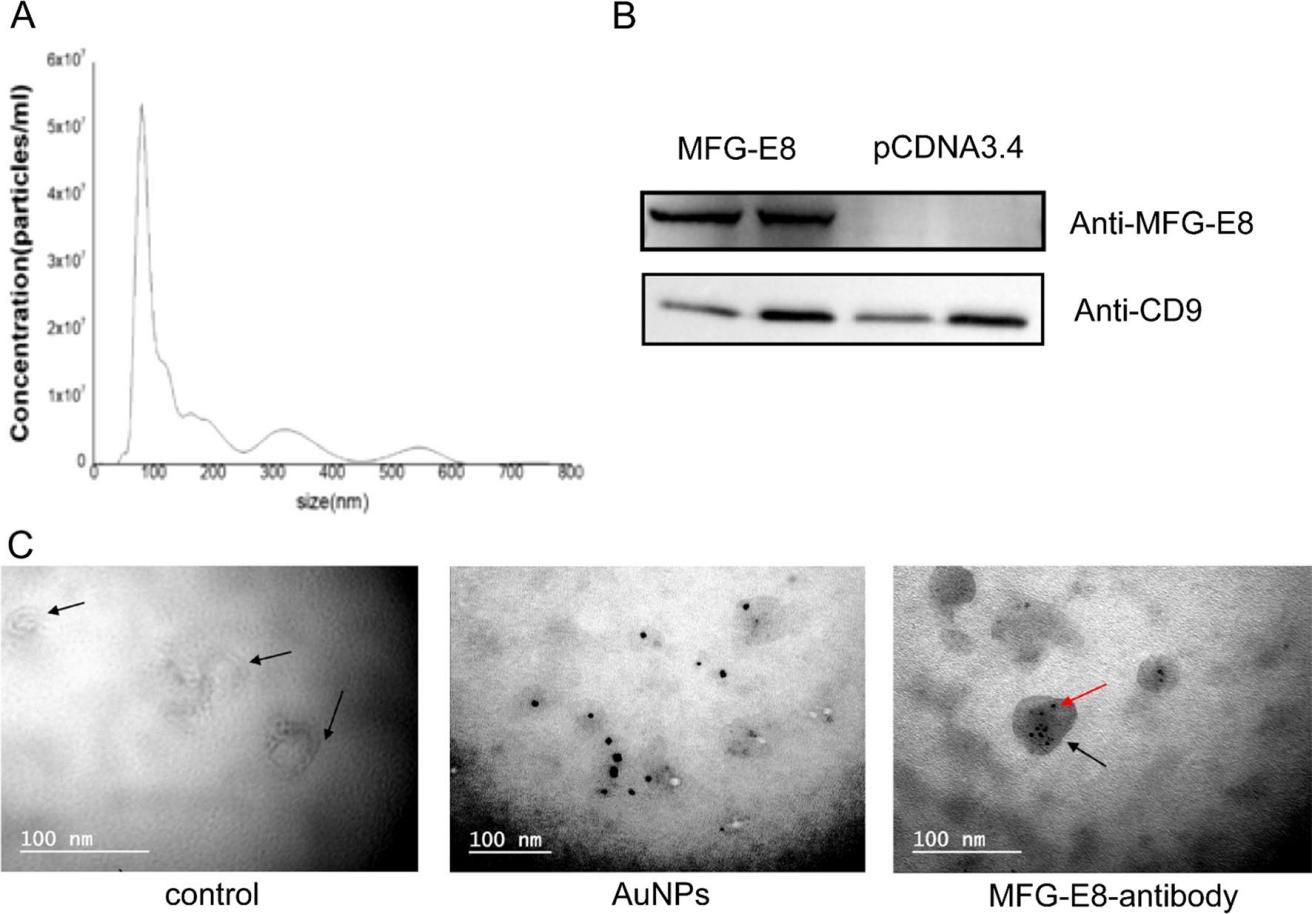
Isolation of sEVs and confirm the MFG-E8 is present outside the sEVs. sEVs were isolated from cell culture on day 4 after transient transfection of recombinant MFG-E8 and were analyzed by NTA analyzer (**a**), and western blotting (**b** and Additional file 1: Figures S4, S5) to confirm the existence of MFG-E8 in sEVs. Under the transmission electron microscopy, MFG-E8 was shown by anti-MFG-E8-AuNPs-mAb (black dots). Black dots, black arrows and red arrows refer to anti-MFG-E8-AuNPs-mAb, sEVs and MFG-E8, respectively.(**c**) (scale bar = 100 nm)

Nanoparticle-tracking analysis (NTA) showed that most of the vesicles in precipitate had a size of 120 nm approximately, corresponding to the range of described sEVs (Fig. 2a). The existence of sEVs was further confirmed by exosomal protein marker CD9 in these vesicles. We also observed MFG-E8 in these sEVs (Fig. 2b). Although MFG-E8 was an exosomal protein, the concentration of it in sEVs from blank HEK 293F cells was lower than that in sEVs secreted from donor cells with overexpression of MFG-E8.

In order to investigate the location of MFG-E8 in sEVs, we examined sEVs under transmission electron microscopy (TEM) after incubating sEVs with anti-MFG-E8 antibodies labeled AuNPs (anti-MFG-E8-AuNPs-mAb). Both the control sEVs and MFG-E8 loaded sEVs exhibited cup-shaped bilayer membranes and were measured at about 100 nm in diameters. Additionally, the anti-MFG-E8-AuNPs-mAb was shown as the black dots under TEM. Because the anti-MFG-E8-AuNPs-mAb was too large to enter into sEVs, it could bind with MFG-E8 only when it was outside the membrane of sEVs, shown as the black dots under TEM. We found that the black dots existed in the sEVs, indicating that MFG-E8 should be located on the outside of the sEVs (Fig. 2c).

### Fusion of MFG-E8 to target proteins results in efficient loading into sEVs

**Fig. 3:**
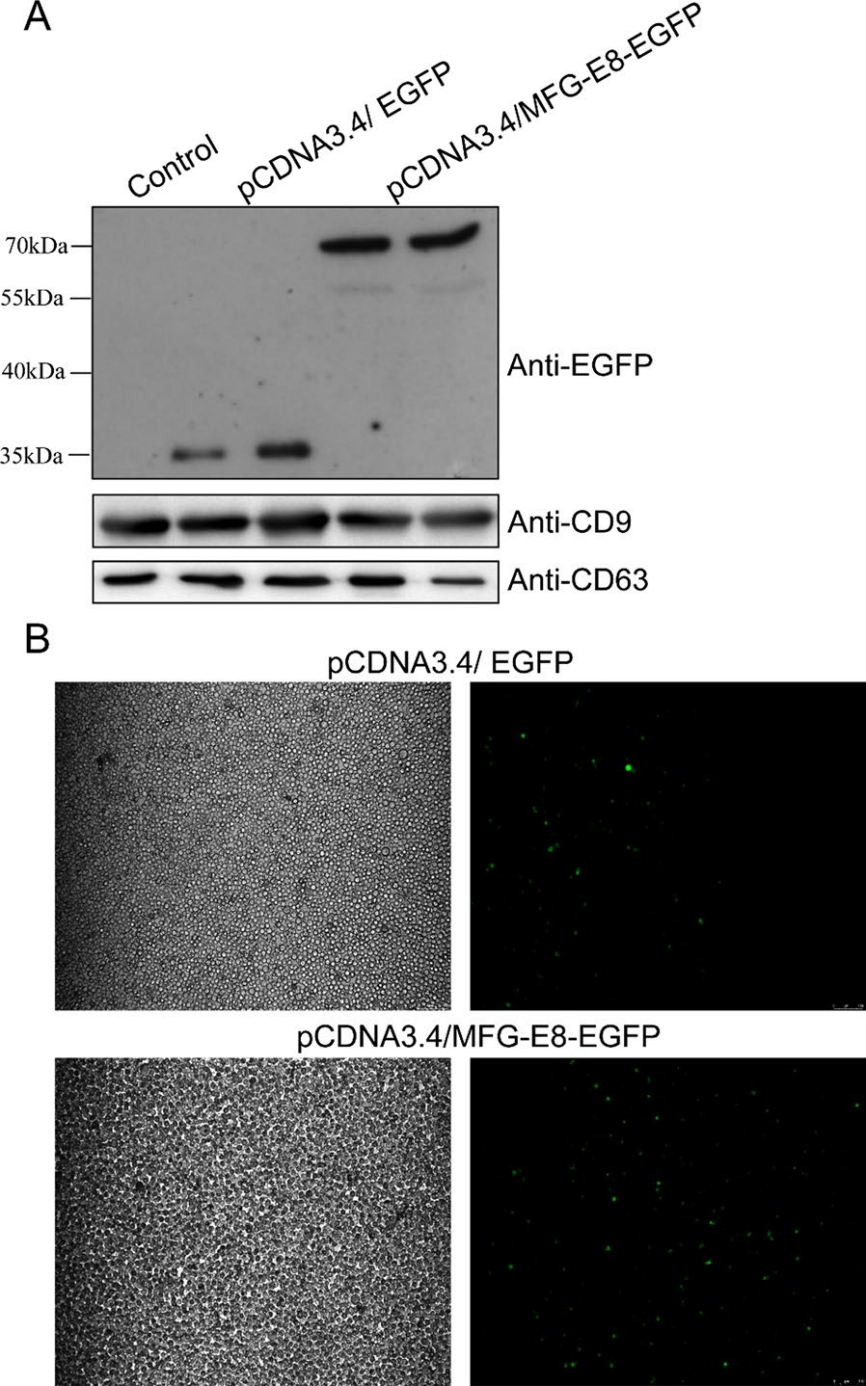
Delivery of EGFP into sEVs by MFG-E8 fusion expression. The sEVs from cells transfected with pCDNA3.4/EGFP or pCDNA3.4/MFG-E8-EGFP were analyzed by western blotting to confirm whether MFG-E8 could address other proteins into sEVs(**a** and Additional file 1: Figures S6, S7, S8). To identify the transfection efficiency of sEVs, the blank HEK293F cells were transfected with sEVs containing EGFP or MFG-E8-EGFP for 6 h and investigated under confocal microscopy (**b**)

In order to investigate if MFG-E8 could address other proteins into sEVs, we transfected HEK293F cells with pCDNA3.4/MFG-E8-EGFP to express this fusion protein (MFG-E8-EGFP), meanwhile using pCDNA3.4/ EGFP as control. sEVs were isolated on day 4 of post-transfection as the aforementioned way. The EGFP were found in both sEVs secreted from HEK293 cells transfected with pCDNA3.4/MFG-E8-EGFP and pCDNA3.4/ EGFP. The EGFP in sEVs secreted from cells transfected with pCDNA3.4/MFG-E8-EGFP have higher molecular weight and higher concentration than that in sEVs from cells transfected with pCDNA3.4/EGFP (Fig. 3a). Although the mechanism of protein sorting into sEVs remained unclear, MFG-E8 could address more EGFP into sEVs by fusion expression (Fig. 3a).

Since the exosomal membrane had the same component as the cell membrane, sEVs could automatically fuse with the membrane of target cells to transfer its contents into the recipient cells. sEVs were thought to be a good natural transfection reagent and drug carrier because of their high transfection efficiency and good biocompatibility. To confirm this, HEK 293F cells were incubated with sEVs loaded with MFG-E8-EGFP. At 6 h of post-transfection, the fluorescence of EGFP was found in recipient cells under confocal microscopy (Fig. 3b).

The results above showed MFG-E8 could address other proteins into sEVs, and then could mediate protein delivery to recipient cells by the transfection of sEVs.

### The target protein delivered into sEVs by MFG-E8 remained its activity

Although MFG-E8 could carry the target proteins into sEVs in a protein fusion manner, whether the proteins in sEVs were active or not could not be demonstrated by the above-mentioned experiment. Therefore, we proceeded to test if proteins remained active after being delivered into sEVs. Gaussia Luciferase (GL) was chosen to be a reporter protein because it could catalyze its substrate to emit fluorescence only when luciferase is active. To achieve this goal, the plasmid pCDNA3.4/MFG-E8-GL was constructed, while pCDNA3.4/CD9-GL was used as a positive control because CD9 is a known exosomal protein that usually served as a protein carrier into sEVs.

**Fig. 4:**
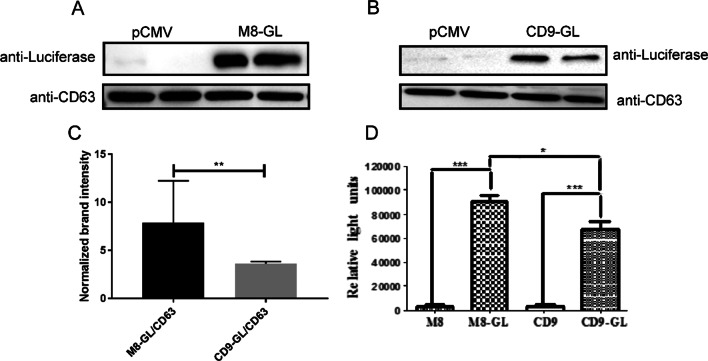
Delivery of Gaussia Luciferase (GL) into sEVs by fusion expression. HEK293F cells were transfected with pCDNA3.4/MFG-E8-GL and pCDNA3.4/CD9-GL respectively and sEVs were isolated. Both M8-GL (**a** and Additional file 1: Figures S9, S11) and CD9-GL (**b** and Additional file 1: Figures S10, S11) were found in the sEVs by western blotting analysis. The relative GL concentration in sEVs was analyzed by comparing M8-GL/CD63 and CD9-GL/CD63 (**c**). And the luciferase activity in two sEVs was compared by analyzing the catalytic activity of the enzyme (**d**)

HEK293F cells were transfected with these two plasmids respectively, and sEVs were isolated in the aforementioned way. MFG-E8-GL(M8-GL) and CD9-GL were confirmed in these two kinds of sEVs using Gaussia luciferase antibodies (Fig. 4a, b). With CD63 as a reference protein of sEVs, we compared relative GL protein concentration in sEVs (GL/CD63). The average value of M8-GL/CD63 (7.52) was significantly higher than CD9-GL/CD63 (3.2) (p < 0.01), indicating MFG-E8 had higher efficiency to deliver target proteins into sEVs (Fig. 4c). Finally, we detected the luciferase activity based on the catalytic activity of its substrate coelenterazine. It was shown that both M8-GL and CD9-GL in sEVs were active to catalyze coelenterazine, the activity of M8-GL was higher than CD9-GL (Fig. 4d). All the above demonstrated the target protein delivered into sEVs by MFG-E8 remained its activity.

### sEVs with MFG-E8 had αvβ3 targeting

**Fig. 5:**
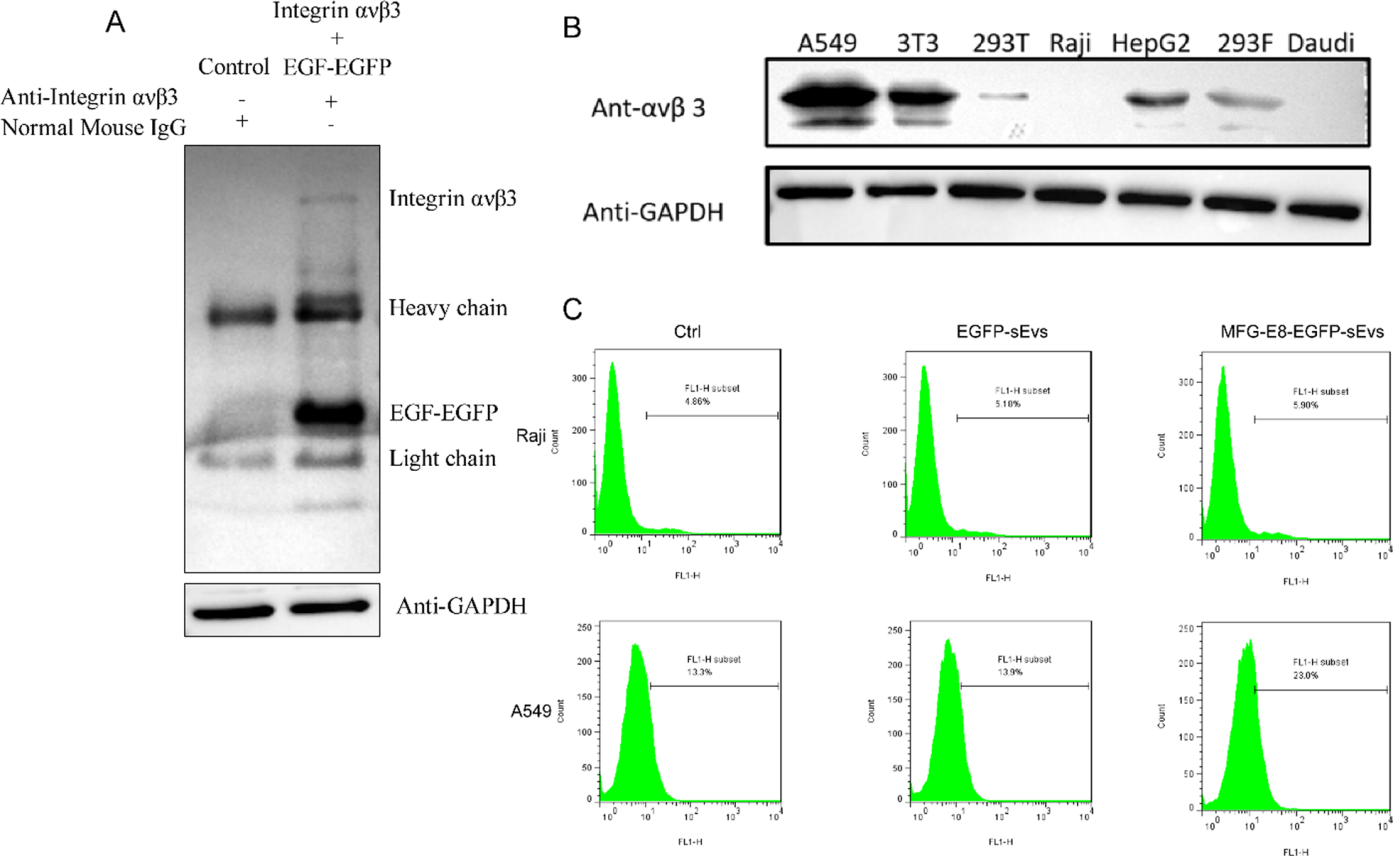
The αvβ3 targeting of sEVs with MFG-E8. The binding between integrin ανβ3 on A549 cell membrane and EGF-like domain of MFG-E8(EGF-EGFP) was confirmed by the immunoprecipitation assay (**a** and Additional file 1: Figures S12, S13).And the αvβ3 expression of several kinds of cells was screened by western blotting (**b** and Additional file 1: Figures S14, S15). After the transfection of sEVs, the concentration of EGFP in A549 cells and Raji cells was analyzed by flow cytometry (**c**). Ctrl, EGFP-sEVs and MFG-E8-EGFP-sEVs refer to cells incubated with blank sEVs, EGFP and MFG-E8-EGFP loaded sEVs, respectively

There was an Arg-Gly-Asp sequence in NH2-terminal epidermal growth factor (EGF)–like domains of MFG-E8, which could bind with αvβ3 and αvβ5 integrins that were usually overexpressed in some tumor cells. Through the immunoprecipitation assay, it comfirmed that the integrin ανβ3 on A549 cells could bind with the EGF-like domain of MFG-E8 (Fig. 5a).

Meanwhile, we screened several types of cells to find integrin αvβ3-positive and αvβ3-negative cells. It was shown that A549, human lung adenocarcinoma cells, had the highest αvβ3 expression, and human lymphoblastoid cells, Raji and Daudi, were αvβ3-negative (Fig. 5b). In order to identify whether the sEVs with MFG-E8 could target integrin αvβ3 because of MFG-E8 outside the exoxomal membrane, A549 cells and Raji cells were chosen as αvβ3-positive and αvβ3-negative cells, respectively. These two kinds of cells were transfected by sEVs containing MFG-E8-EGFP(MFG-E8-EGFP-sEVs), using sEVs containing EGFP(EGFP-sEVs) as control. Analysis by flow cytometry showed about 9.7% of A549 cells were EGFP-positive while only 1.04% of Raji cells were EGFP-positive. Compared to the cells transfected with EGFP-sEVs, those transfected by MFG-E8-EGFP-sEVs had more EGFP transferred into the cells (Fig. 5c). The above results indicated that sEVs not only could carry target proteins into sEVs, but also make sEVs could target αvβ3 in recipient cells.

## Discussion

Therapeutic proteins and polypeptides, such as enzymes, cytokines, and antibodies, are available for treating various human diseases. However, these protein-based drugs usually are sensitive to changes in temperature, solvent, and pH, posing significant challenges in achieving the best therapeutic outcomes. Moreover, the majority of clinically available biopharmaceutical drugs are limited to the extracellular environment because of their poor membrane permeation [34, 39]. With a small size and the same bilayer cellular membrane as human cells, sEVs are promising drug carriers although their application was hindered by lacking efficient methods of cargo loading. For protein-based therapeutics, endogenously loading has been exploited in several reports by fusion or interaction with proteins enriched in sEVs, one of which is MFG-E8 [37, 38].

MFG-E8, also called lactadherin, was originally identified as a component of milk fat globules that bud from the mammary epithelial and later on was found in many kinds of cells [40]. MFG-E8 has an epidermal growth factor (EGF)–like domains at NH2 –terminal, which contain a conserved arginine-glycine-aspartate (RGD) motif that can bind with αvβ3 and αvβ5 integrins, and C1C2 domain at COOH terminal domains, which could bind with phospholipids, especially phosphatidylserine [41]. MFG-E8 can act as a bridge between apoptotic cells and macrophages by binding with the PS of apoptotic cells through its C1C2 domain and also attaching to the αvβ3/αvβ5-integrin expressed on activated macrophages through the RGD motif [42]. In our study, we found that MFG-E8 could be secreted from cells and attached to the cell membrane, but not into the media.

Because of its adhesion to the membrane, the C1C2 domain can be used to target other proteins or peptides onto sEVs [36, 43]. When the C1C2 domain was fused with other proteins such as interleukin 2 (IL-2) or granulocyte–macrophage colony-stimulating factor (GM-CSF) instead of the EGF-like domain, the fusion proteins were found in sEVs secreted by cells [43]. In our study, fusion with the whole MFG-E8 also addressed other proteins to the sEVs. By TEM analysis, we found the fluorescence of EGFP (the fusion expression of MFG-E8-EGFP) circled the outside of sEVs, meaning the fusion protein located on the surface of sEVs. Zeelenberg IS et al. reported that sEVs with tumor antigens addressed by fusion with the C1C2 domain could induce efficient antitumor immune responses [36]. All of these indicated that the fusion of peptides to the MFG-E8 or C1C2 domain could be used to display peptides or proteins on the surface of sEVs. However, the activity of the addressed protein or peptides by MFG-E8 or C1C2 domain may be inhibited because of its close association with the membrane, so we further identified the protein activity by fusion expression of MFG-E8 with luciferase. Unlike EGFP, there was fluorescence emitted only when luciferase catalyzes its substrate. It was found the protein fused with MFG-E8 could remain its activity.

Besides sEVs' unique possibilities for cargo loading, sEVs may also offer beneficial features for drug delivery in terms of targeting. In contrast with synthetic lipid nanoparticles, whose stability would be affected by the addition of targeting peptides and whose synthesis is complicated, displaying targeting ligands on sEVs is relatively simple because peptide ligands can be genetically fused to the extra-exosomal termini of exosomal membrane proteins [33]. sEVs targeting specifically neurons, microglia, and oligodendrocytes in the brain have been achieved by engineering dendritic cells and HEK293 cells to fusion express the neuron-specific rabies viral glycoprotein(RVG) peptide and an exosomal membrane protein Lamp2b [23, 44]. Similarly, the RGD peptide was another targeting peptide that was used to engineer the sEVs to target breast cancer cells via αvβ3 integrin by fusing to the N terminus of Lamp2b [45]. Because there is an RGD motif in MFG-E8, we supposed that the engineered sEVs with MGF-E8 would have the capability of targeting αvβ3 integrin. Our study demonstrated that sEVs engineered with MFG-E8 could deliver more protein of interest into receptor cells, and more proteins could transfect into αvβ3-positive cells than αvβ3-negative cells by the transfection of engineered sEVs, meaning MFG-E8 engineering could facilitate sEVs αvβ3 integrin targeting.

In our study, we chose to transfect HEK293F cells with MFG-E8-expressing plasmid to acquire an engineered sEVs. Through fusion expression of MFG-E8 and the proteins of interest, we demonstrated that MFG-E8 is not only a suitable delivery protein that can address other proteins to sEVs, but also able to confer sEVs the targeting capabilities to high integrin cells such as some tumor cells. It has also been reported that MFG-E8 may play a positive role in the membrane secretion to improve the sEVs budding [37, 38], although, in our study, transfection of HEK293 cells with MFG-E8 only increased sEVs slightly, but not significantly. In summary, MFG-E8 is a suitable protein with both abilities of cargo loading and sEVs targeting integrin αvβ3.

## Conclusions

According to the results of the present study, recombinant MFG-E8 could be secreted and adhere to the outside of the HEK293F cell membrane. By expressing protein fused to MFG-E8, the delivery efficiency of the interest protein into sEVs, then into recipient cells, could be increased. Furthermore, MFG-E8 could not only encapsulate active protein in sEVs but also make sEVs could target αvβ3 in recipient cells.

## Methods

### Cell culture

For the suspension culture, HEK293F cells (ATCC, ACS-4500™) were cultivated in 50-mL Tubespin containing 10 mL ProCHO5 medium (Lanza Co.) at a density of 0.5 × 10^6^cells/mL. Cultures were maintained in a shaking incubator at 37 °C with a stirring speed of 180 rpm. For adherent cell culture, HEK293T cells (ATCC, CRL-11268™) were incubated in 5 mL DMEM medium containing 10% fetal calf serum (Gibco) in incubator at 37 °C. Raji cells(CCL-86™), and A549 cells(CRM-CCL-185™) were incubated in 5 mL RPIM 1640 medium containing 10% fetal calf serum (Gibco) in incubator at 37 °C. The cell density and viability were determined by the Trypan Blue exclusion method.

### Plasmids

All plasmids were constructed with plasmid pCDNA3.4 (Invitrogen co.), including pCDNA 3.4/MFG-E8, pCDNA3.4/MFG-E8-EGFP, pCDNA3.4/EGFP, pCDNA3.4/MFG-E8-Gaussia luciferase(GL), and pCDNA3.4/CD9-GL. After digestion with KpnI and XhoI, the open reading frame (ORF) of MFG-E8, EGFP, MFG-E8-GL, or CD9-GL was inserted into the multiple cloning site (MCS) of pCDNA3.4. For fusion expression, a flexible linker (GGGGSGGGGSGGGGS) was used to link DNA sequences of two genes.

### Transient expression of protein in HEK293F cells

One day prior to transfection, HEK 293F cells were seeded in fresh ProCHO5 medium at a density of 2 × 10^6^ cells/mL. On the day of transfection, cells were centrifuged at 800 rpm for 5 min and resuspended in 2 mL RPMI1640 media at the indicated cell density in TubeSpins. The plasmid DNA and 25 kDa linear polyethyleneimine (PEI, Polysciences, Warrington, PA) were mixed and stood for 10 min, and then added to the culture. The transfected culture was incubated for 3 h at 37 °C with 5% CO2, 85% humidity, and agitation at 180 rpm, followed by adding EX-Cell HEK293 medium (Sigma) to 10 mL.

### sEVs isolation

Cell culture medium was collected and centrifuged at 3,000×g for 15 min to remove cellular debris, and the supernatants were transferred to an appropriate vessel for the CP70ME ultracentrifuge (Hitachi, Ltd. Japan) according to the method described by Théry C [46]. Successive centrifugations at increasing speeds were performed to throw the pellet away (300×g for 10 min-2000×g for10 min–10,000×g for 30 min). In the last step, the supernatant was collected and centrifuged one more time at 100,000×g for 70 min and only the pellets were kept. The pellet was washed in a large volume of PBS three times to eliminate contaminating proteins and centrifuged at the same high speed. The final sEVs pellets were resuspended in 100 mL PBS and filtered through a syringe filter (0.2 mm, Sartorius). The morphology of sEVs was observed by Transmission Electronic Microscopy (FEI co., CZ). The number and size of sEVs particles were measured by nanoparticle-tracking analysis with Nanosight NS300 (Malvern Instruments).

### Western blotting

RIPA buffer was added to cells for incubation at 4°C for 10 min, then the supernatant(cells lysates) was collected by centrifugation. Cell lysates and isolated sEVs were subjected to 12% SDS-PAGE electrophoresis and western blotting according to standard protocols. After electrophoresis, the proteins are transferred from the gel to a nitrocellulose filter membrane by electrotransfer. The nitrocellulose filter membrane was incubated at 4 °C for 16 h with the indicated primary antibodies against MFG-E8, EGFP, CD9, CD63, luciferase, or αvβ3 (Invitrogen, Cat.No. PA5-82036; Proteintech, Cat.No. 66002–1-Ig; 60232–1-Ig; 67605–1-Ig; 67293–1-Ig; 66952–1-Ig; Abcam, Cat. No. ab190147) and then washed for three times in Tris-buffered saline T (TBS-T), followed by 1 h incubation with Goat Anti-Mouse IgG(H + L) (Proteintech, Cat.No. SA00001-1) at room temperature.

### Confocal microscopy

Cells were successively incubated with 4% paraformaldehyde, 0.25% TritonX-100, and 1% BSA. At the end of each step, cells were centrifuged and washed with PBS buffer. In the end, cells were incubated with anti-MFG-E8 antibodies solution for 12 h at 4 °C, followed by incubation with Goat Anti-Mouse IgG H&L (FITC) (Abcam, Cat.No. ab6785) and DAPI solution respectively, then observed by Zeiss laser confocal microscopy.

### Transmission Electronic Microscopy (TEM) analysis of gold nanoparticles labeled sEVs

The preparation of gold nanoparticles (AuNPs) and gold nanoparticles labeled mAb (AuNPs-mAb) was done according to reference [47]. Briefly, 1 mL of 1% HAuCl4 was quickly added to the 50 mL boiled ultrapure water and 1.2 mL trisodium citrate dihydrate (10 mg/mL) was added after a few seconds. The mixture was heated for 10 min and then diluted with ultrapure water to 50 mL.

For preparation of AuNPs-antibody, 1 mL of AuNPs (0.02 mg/mL) was adjusted to pH 8.5 with 0.25 M K_2_CO_3_, and 10 μL anti-MFG-E8 antibody was diluted with 1 × TBST to 100 μL. Then the antibody was quickly added to the AuNPs solution. The mixture was rotated for 15 min and kept still for 15 min at room temperature. Subsequently, 100 μL of 10% BSA was added to cover the unconjugated site, rotated for another 15 min, then kept still for 15 min. Finally, the mixture was centrifuged at 12,000 rpm for 30 min and the precipitate was resuspended in 50–100 μL PBS, followed by incubation with isolated sEVs overnight at 4 °C. 10 μL of labeled sEVs was dropped on GRID and applied to TEM after air-drying.

### Gaussia Luciferase (GL) activity analysis

The sEVs loaded M8-GL or CD9-GL were rinsed with 100 μL/well of 1×PBS buffer,  then RIPA buffer was added to the sEVs for incubation at 4°C for 10 min, followed by centrifugation to collect the supernatant(sEVs lysate).

Gaussia Luciferase (GL) activity was analyzed according to the protocol Pierce™ Gaussia Luciferase Glow Assay Kit (Thermo Scientific). In brief, the Working Solution was prepared by adding 50 μL of 100×coelenterazine to 5 mL of Gaussia Glow Assay Buffer first. 10–20 μL/well of sEVs lysate was added to an opaque 96-well plate, then 50 μL of Working Solution was added to each well. After 10 min of signal stabilization, the light output was detected in a Luminometer (Berthold, Bad Wildbad, Germany).

### Flow cytometry

To confirm whether recombinant MFG-E8 adhered to the cell membrane, HEK 293F cells were transfected with pCDNA3.4/MFG-E8  and cultured in incubator at 37 °C with 5% CO_2_. On day 4 of post-transfection, 1×10^6^ cells were placed in a 1.5 mL tube, then centrifuged at 1000×g for 5 min and washed three times with 1 × PBS. Next, the cells were resuspended in 100 μL PBS, and 5 μL of the anti-MFG-E8 antibody was added to the cell suspension. The sample was mixed thoroughly at 37 °C in the dark for 30 min, followed by centrifugation and washing again. Finally, 5 μL anti-Mouse IgG H&L(FITC) was added to the cell suspension, and the sample was mixed again at 37 °C in the dark for 30 min. After centrifuged at 1000×g for 5 min and washed three times with 1 × PBS, the cells were resuspended in 500 μL of PBS and examined using flow cytometry. To demonstrate sEVs with MFG-E8 had αvβ3 targetings, cells were placed in a 1.5 mL tube after incubating with MFG-E8-EGFP-sEVs and EGFP-sEVs, then contrifugated and detected under cytometry as abovementioned way.

### Immunoprecipitation assay

First, HEK 293F cells were transfected to express EGF-like domain of MFG-E8 as abovementioned way. Secondly, the recombinant HEK 293F cells and A549 cells were collected, and RIPA buffer was added separately for incubation at 4°C for 10 min. Then both of the supernatant was collected after centrifugation at 10,000×g for 10 min at 4 °C and transferred to a fresh centrifuge tube, followed by adding of 20 μL Protein A/G PLUS-Agarose and 1.0 μg Goat Anti-Mouse IgG(H+L) as control. After incubation at 4 °C for 30 min, the supernatant were collected by centrifugation at 2500 rpm for 5 min at 4 °C, and 1mL of supernatant was transferred to a fresh tube on ice, followed by addition of 1–10 μL Integrin Beta 3 Monoclonal antibody(Proteintech, Cat.No. 66952-1-Ig) to incubat for 1 h at 4 °C, then further addition of 20 μL Protein A/G PLUS-Agarose for incubation at 4 °C on a rocker platform overnight. After a night, the immunoprecipitates were collected by centrifugation at 2500 rpm for 5 min at 4 °C. Finally, the pellet was washed 4 times with PBS and analyzed by western blotting.

## Supplementary Information

**Additional file 1**. Original pictures.

## Data Availability

The datasets supporting the conclusions of this article are included with in the article. All strain materials were obtained from Jinan University, Guangzhou, China. The sequences used during the current study are available in the NCBI repository https://www.ncbi.nlm.nih.gov/nuccore/NM_005928.4, https://www.ncbi.nlm.nih.gov/nuccore/LC006266.1, https://www.ncbi.nlm.nih.gov/nuccore/AH006868.3, https://www.ncbi.nlm.nih.gov/protein/AFA52654.1.

## Abbreviations

sEVs: Small extracellular vesicles
MFG-E8: Milk fat globule–epidermal growth factor 8 protein
EGF: Epidermal growth factor
TEM: Transmission electron microscopy
NTA: Nanoparticle-tracking analysis
GL: Gaussia Luciferase
