# Supplementary material for: αvβ3-targeted sEVs for efficient intracellular delivery of proteins using MFG-E8
Source: BMC Biotechnol. 2022 May 21;22:15. doi: 10.1186/s12896-022-00745-7 (PMC9123705; doi:10.1186/s12896-022-00745-7)
Supplement: Supplementary file 1 — Additional file 1. Original pictures. [file 12896_2022_745_MOESM1_ESM.pdf]

Original pictures

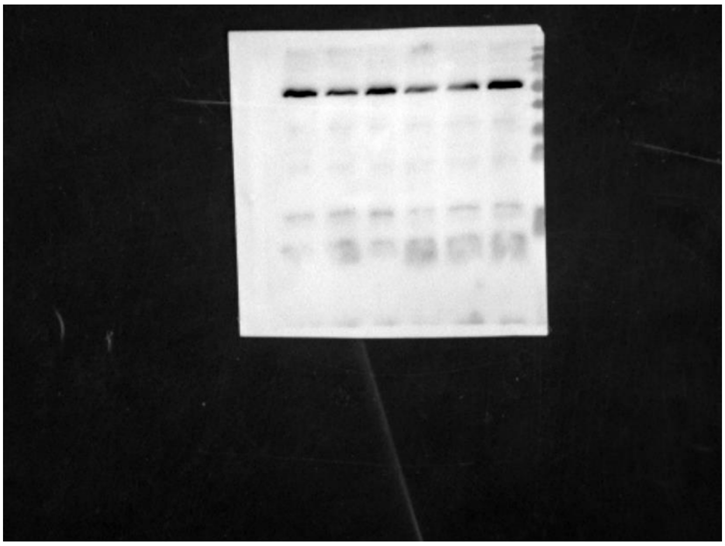

Figure S1-Anti-MFG-E8

Figure S1 is the original picture of Fig. 1a. The expression of recombinant MFG-E8 was confirmed by Western blot.

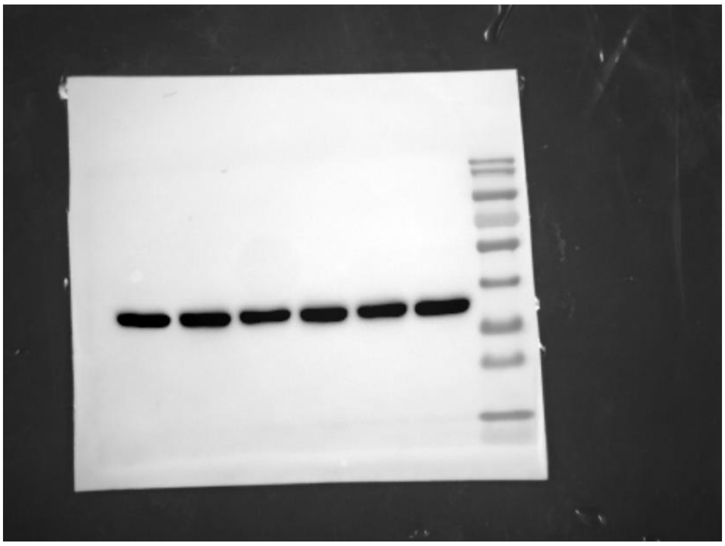

Figure S2-GAPDH

Figure S2 is the original picture of Fig. 1a. The expression of GADPH was confirmed by Western blot.

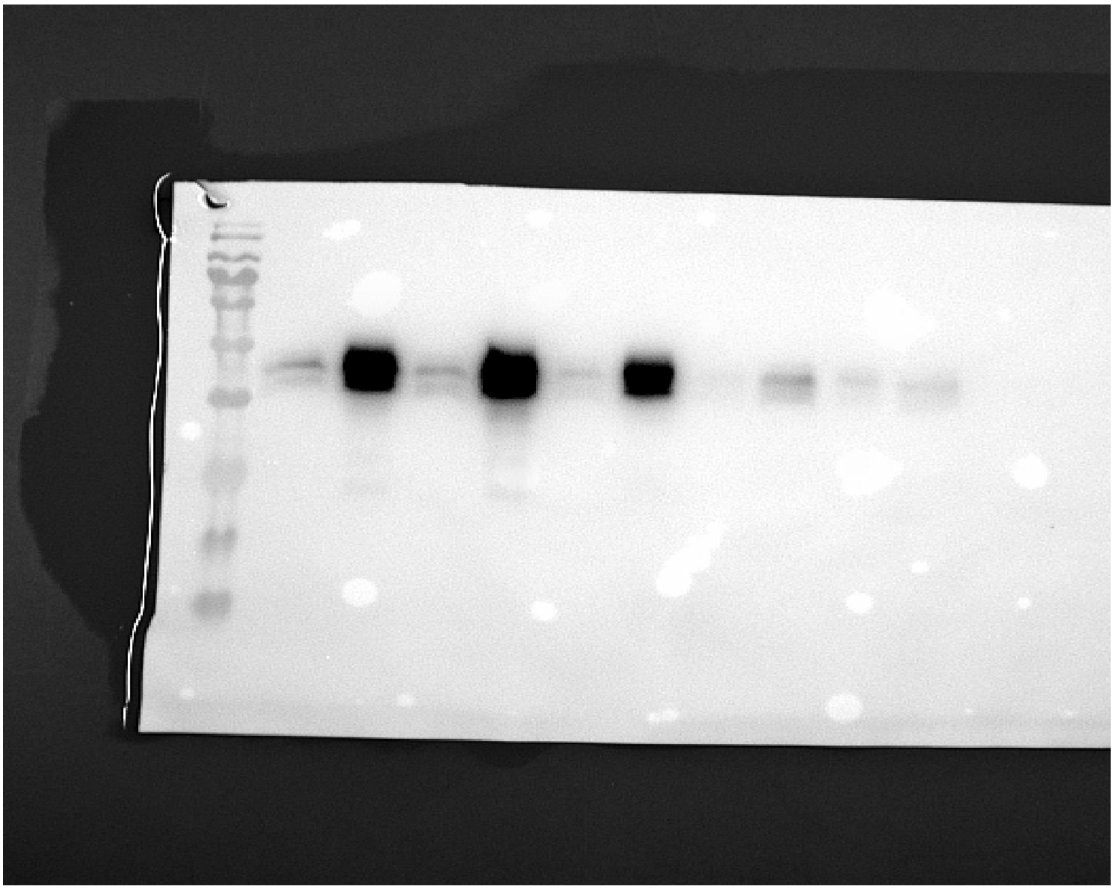

Figure S3-Anti-MFG-E8

Figure S3 is the original picture of Fig. 1b. The expression of MFG-E8 was confirmed by Western blot.

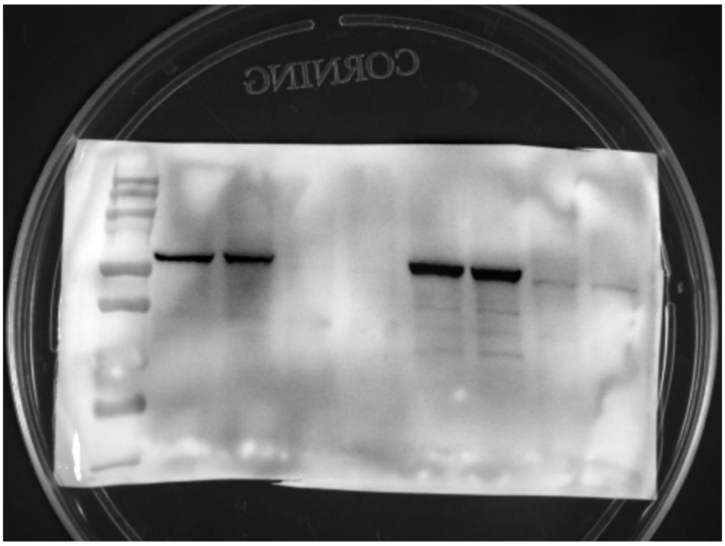

Figure S4-Anti-MFG-E8

Figure S4 is the original picture of Fig. 2b. The expression of MFG-E8 was confirmed by Western blot.

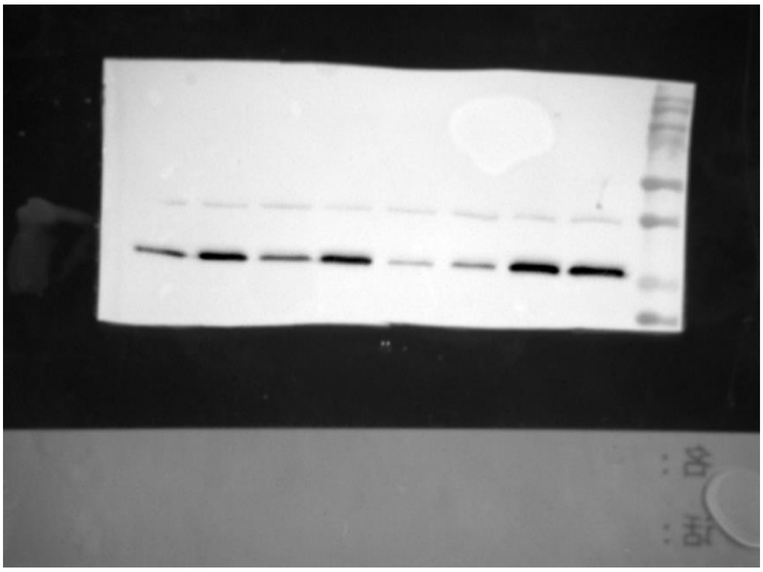

Figure S5-Anti-CD9

Figure S5 is the original picture of Fig. 2b. The expression of CD9 was confirmed by Western blot.

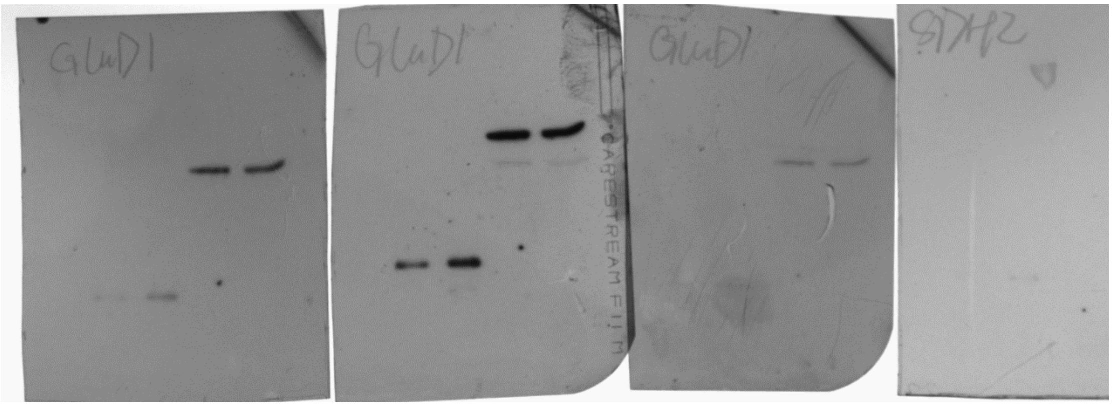

Figure S6-Anti-EGFP

Figure S6 is the original picture of Fig. 3a. The expression of EGFP and MFG-EGFP was confirmed by Western blot.

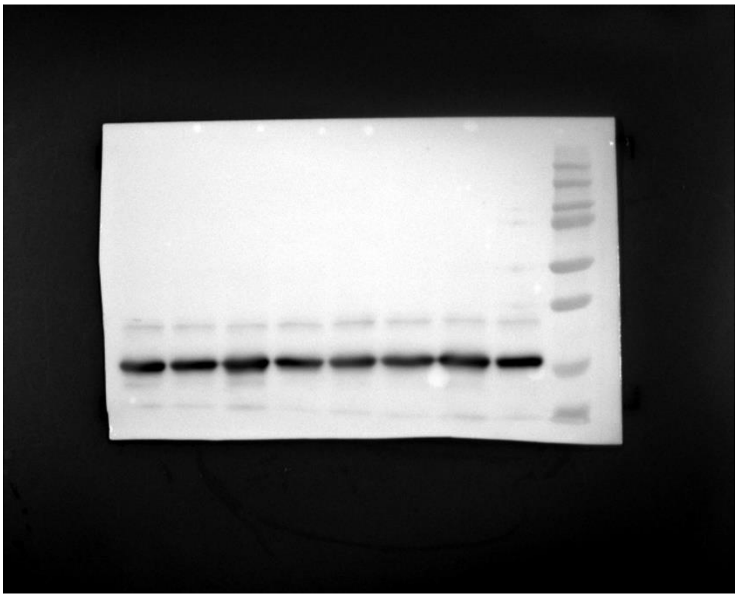

Figure S7-Anti-CD9

Figure S7 is the original picture of Fig. 3a. The expression of CD9 was confirmed by Western blot.

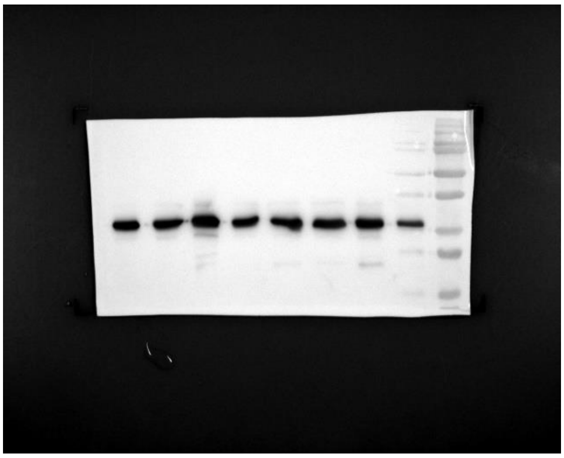

Figure S8-Anti-CD63

Figure S8 is the original picture of Fig. 3a. The expression of CD63 was confirmed by Western blot.

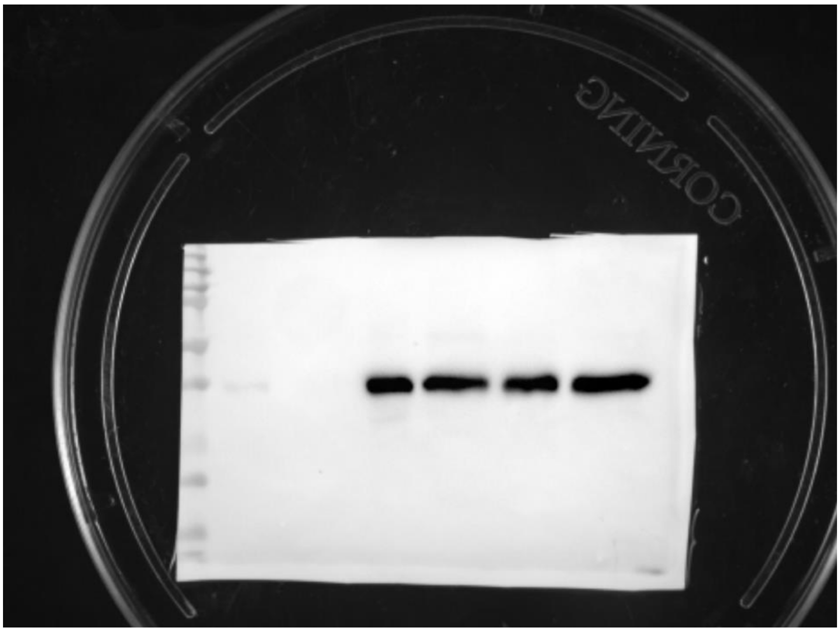

Figure S9-M8-GL-anti-Luciferase

Figure S9 is the original picture of Fig. 4a. The expression of M8-GL was confirmed by Western blot.

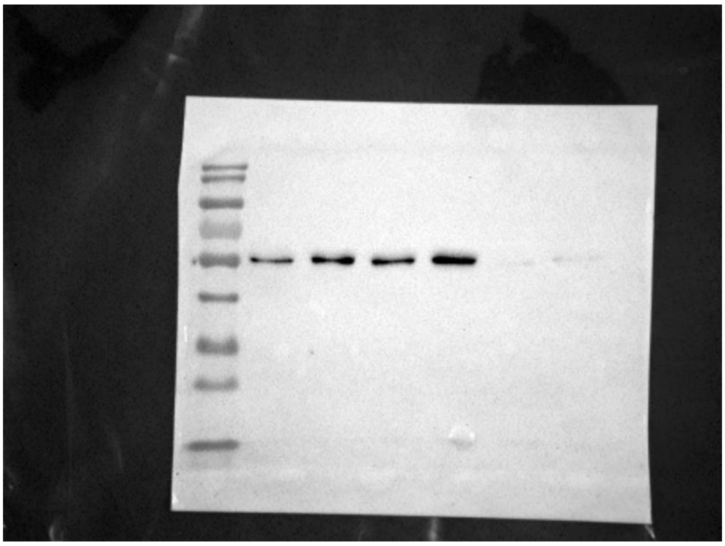

Figure S10-CD9-GL- anti-Luciferase

Figure S10 is the original picture of Fig. 4b. The expression of CD9-GL was confirmed by Western blot.

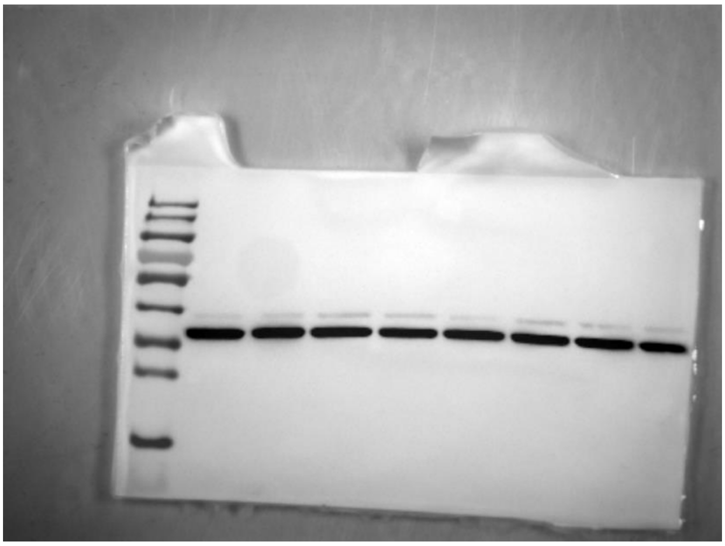

Figure S11-anti-CD63

Figure S11 is the original picture of Fig. 4ab. The expression of CD63 was confirmed by Western blot.

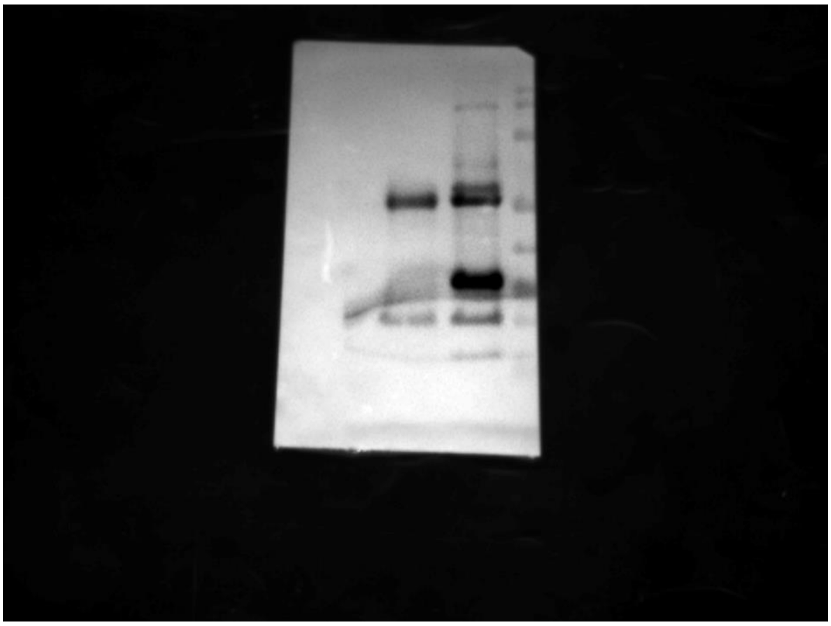

Figure S12-IP-αvβ3+EGF-EGFP

Figure S12 is the original picture of Fig. 5a. After immunoprecipitation assay, integrin αvβ3 and EGF-EGFP were confirmed by Western blot.

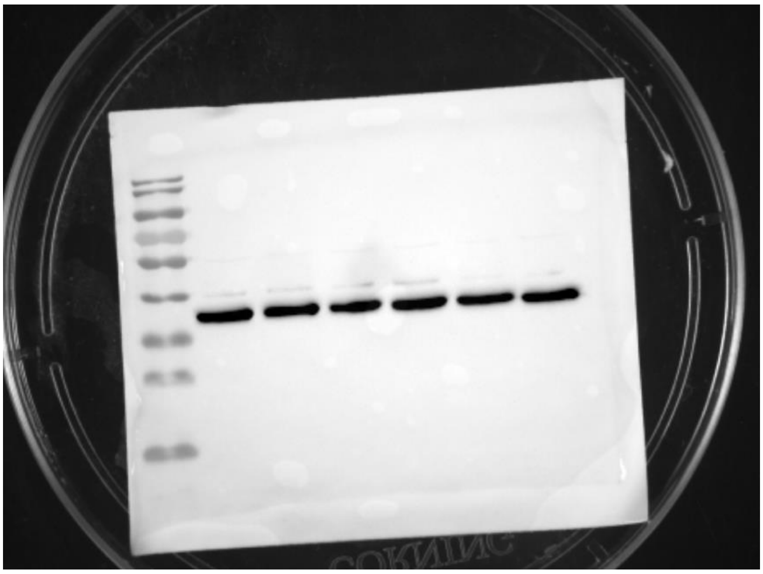

Figure S13-Anti-GAPDH

Figure S13 is the original picture of Fig. 5a. The expression of GAPDH was confirmed by Western blot.

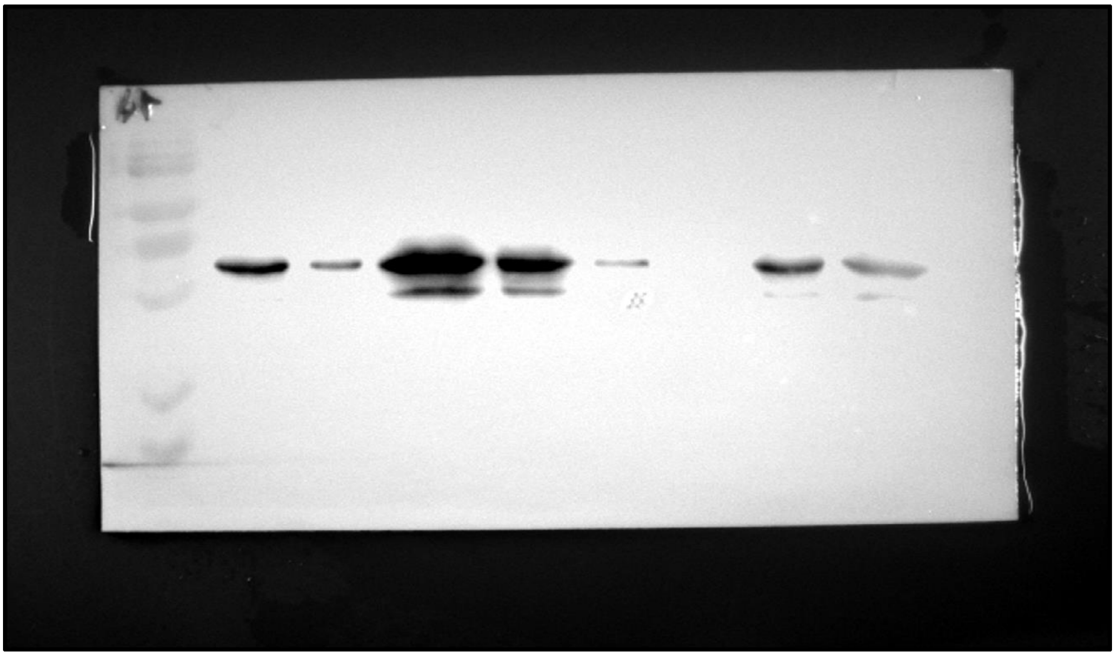

Figure S14-Anti-αvβ3

Figure S14 is the original picture of Fig. 5b. The expression of integrin αvβ3 was confirmed by Western blot.

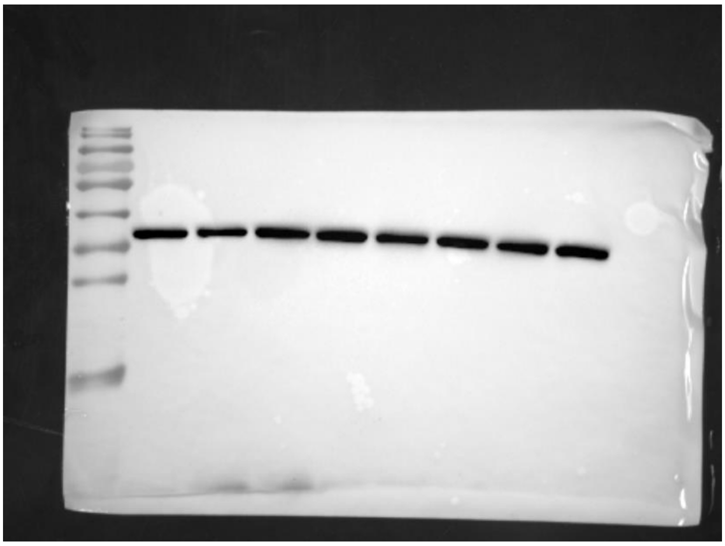

Figure S15-anti-GAPDH

Figure S15 is the original picture of Fig. 5b. The expression of GAPDH was confirmed by Western blot.
